# NMR-based metabolomics of plasma from dairy calves infected with two primary causal agents of bovine respiratory disease (BRD)

**DOI:** 10.1038/s41598-023-29234-3

**Published:** 2023-02-15

**Authors:** Mariana Santos-Rivera, Nicholas C. Fitzkee, Rebecca A. Hill, Richard E. Baird, Ellianna Blair, Merrilee Thoresen, Amelia R. Woolums, Florencia Meyer, Carrie K. Vance

**Affiliations:** 1 Department of Biochemistry, Molecular Biology, Entomology, and Plant Pathology, Mississippi State University, Mississippi State, MS 39762 USA; 2 Department of Chemistry, Mississippi State University, Mississippi State, MS 39762 USA; 3 College of Veterinary Medicine, Pathobiology and Population Medicine, Mississippi State University, Mississippi State, MS 39762 USA

**Keywords:** Biochemistry, Biological techniques, Biomarkers, Diseases

## Abstract

Each year, bovine respiratory disease (BRD) results in significant economic loss in the cattle sector, and novel metabolic profiling for early diagnosis represents a promising tool for developing effective measures for disease management. Here, ^1^H-nuclear magnetic resonance (^1^H-NMR) spectra were used to characterize metabolites from blood plasma collected from male dairy calves (n = 10) intentionally infected with two of the main BRD causal agents, bovine respiratory syncytial virus (BRSV) and *Mannheimia haemolytica* (MH), to generate a well-defined metabolomic profile under controlled conditions. In response to infection, 46 metabolites (BRSV = 32, MH = 33) changed in concentration compared to the uninfected state. Fuel substrates and products exhibited a particularly strong effect, reflecting imbalances that occur during the immune response. Furthermore, ^1^H-NMR spectra from samples from the uninfected and infected stages were discriminated with an accuracy, sensitivity, and specificity ≥ 95% using chemometrics to model the changes associated with disease, suggesting that metabolic profiles can be used for further development, understanding, and validation of novel diagnostic tools.

## Introduction

One of the most severe and costly health problems worldwide is bovine respiratory disease (BRD), a disease complex caused by numerous microbial pathogens^1–5^. Prevalent causal agents for BRD include viral (bovine herpes-virus type 1, bovine respiratory syncytial virus, bovine viral diarrhea virus, parainfluenza-3 virus, and bovine coronavirus), and bacterial (*Mannheimia haemolytica, Pasteurella multocida, Haemophilus somnus, Mycoplasma bovis*) pathogens^4,6,7^. Bovine respiratory syncytial virus (BRSV) is a primary cause of respiratory disease in young calves (≤ 1 year). This viral infection can be asymptomatic and can involve the upper and lower respiratory tracts^8,9^. BRSV typically initiates infection in response to physiological and environmental stressors, suppressing the host's defense mechanisms and predisposing the replication, inhalation, and colonization of the lungs by *M. haemolytica,* a microorganism found in the normal flora of the upper respiratory system in ruminants^7,10,11^.

The standard in-field method for BRD detection is the visual-clinical diagnosis (VCD) based on scoring systems that record the clinical signs of illness in cattle such as high temperature, respiratory discomfort, nasal and ocular secretions, and other factors considered to represent respiratory disease^12–17^. In dairy cattle, this methodology was reported to have a diagnostic sensitivity between 77–100%, and a screening sensitivity between 46–77%, meaning that around 23–54% of infections or suspected infected animals are not detected^13,14,18^. In addition, the average specificity of this methodology has been reported between 46–92%, indicating that 8–54% of healthy cattle are unnecessarily treated^13,14,18^. Thus, alternative methods to VCD are needed to detect BRD before its manifestation, which will enable farmers to respond with the proper prophylactic measures^19^. One such example of is the use of Near Infrared Spectroscopy (NIRS), which is a novel approach that may facilitate diagnosis of BRD at earlier time points and in mild or asymptomatic cases by detecting changes in a suite of biomarkers resulting from metabolic changes in response to disease state^15,19–21^. A comprehensive phenotypic assessment for biomarkers of BRD using a specific “omics” platform would need to be obtained from blood plasma or other biofluids. To this end, metabolomics profiling of disease state using nuclear magnetic resonance (NMR) is a method for understanding the biochemical processes that occur throughout infection and their relationship with clinical signs detected by traditional diagnostic approaches (e.g., VCD, ELISA).

NMR-based metabolomics provides a window into metabolic mechanisms by combining high-throughput analytical chemistry with multivariate data analysis (MVA) to identify and quantify changes in metabolic products of a biological system^19,22^. Proton NMR (^1^H-NMR) spectra arise from active nuclei absorbing electromagnetic energy at the frequencies specific to the ^1^H nucleus, resulting in resonance within a two-level quantum system^23^. This resonance frequency, along with the signal intensity, is specific to the local covalent-bonding structure and chemical environment and is reflected in the manifold of information-rich signals (chemical shifts) in NMR spectra^23,24^. Plasma is the most frequently used biofluid for NMR studies^19,20,25^, and consists of the protein-rich fraction of blood in which white blood cells, red blood cells, platelets, and coagulation factors are suspended before blood fractionation with an anticoagulant^26^. This biofluid is commonly used to diagnose viral or bacterial infections by detecting antigens or pathogen-specific antibodies using ELISA (Enzyme-Linked Immuno-Sorbent Assay)^26^. Recently, plasma was shown to be a suitable medium for detecting *M. haemolytica* infection using NIRS^15^.

In cattle, NMR has been used to conduct metabolic profiling in the diagnosis of both reproductive and nutritional disorders^27–32^. In one case, seven plasma metabolites (alanine, arginine, choline, isoleucine, leucine, phosphatidyl choline, and valine) were shown to significantly decrease in dairy cows during estrous compared to cows in anestrous. These changes were related to glucose, triglyceride, and amino acid metabolic pathways associated with postpartum anestrus^27^. Similarly, changes in the concentration of metabolites in plasma were observed in Holstein cows during postpartum and lactation periods, revealing that glucose is rerouted to synthesize lactose and fats in milk, causing the lactating cow to produce ketone bodies as an alternative energy source to maintain homeostasis^28–31^. Metabolic profiles related to fatty liver disease in lactating cows were correlated with increases in β-hydroxybutyric acid, acetone, citrulline, glycine, isobutyrate, trimethylamine-N-oxide, and valine, and decreases in γ-aminobutyric acid glycerol, alanine, asparagine, creatinine, and glucose, suggesting this metabolic disorder alters the concentration of metabolites related to energy imbalance pathways^32^. In contrast, NMR analysis revealed calves with bronchopneumonia detected by VCD exhibited increases in 2-methyl glutarate, phenylalanine, and phosphatidylcholine, but showed decreases in acetate, allantoin, cholesterol, dimethyl sulfone, ethanol, propionate, and free cholesterol in the plasma, suggesting alteration of a different set of metabolic pathways^33^. Recently, feedlot cattle that were deemed to have BRD through VCD inspection were shown to have significant alterations in the concentration of α-glucose chains, hydroxybutyrate, and phenylalanine by NMR analysis of plasma^34^. The results from both of these studies^33,34^, combined with recent work in which NIRS profiling of plasma from calves with induced *M. haemolytica* infection^15^, indicate that characteristic shifts in the metabolome of plasma may be indicative of BRD infection and perhaps pathogenic specificity.

Here, we conduct challenge studies, in which dairy calves were intentionally infected with two of the main BRD causal agents, BRSV and *M. haemolytica* (MH), in order to generate a well-defined metabolomic profile under controlled conditions, including mild or asymptomatic cases. ^1^H-NMR analysis of the collected plasma was used to (1) identify metabolites associated with infection by the two different pathogens, (2) assess concentration changes of those metabolites between the healthy and infected stages and in response to each pathogen, (3) generate a model for discriminating ^1^H-NMR spectra, and the metabolites involved in the differentiation of healthy and infected calves for each causal agent, and (4) provide new biochemical information to the current plasma NMR-BRD metabolome. Previously, samples were acquired based on VCD under field conditions^33,34^. The results from our study provide insight into whether there are quantifiable differences in the metabolic profile in response to different causal agents that might be targeted for the understanding, development, and validation of future BRD diagnostic strategies.

## Materials and methods

### Animals and controlled challenges

Ten healthy non-immunized Holstein steers (Table 1) were acquired in the first week of life from the Mississippi Agricultural and Forestry Experiment Station (MAFES) Bearden Dairy Research Center adjacent to the Mississippi State University campus and raised in isolation until two weeks before the start of the experiments when they were housed in isolation from all other cattle. The calves in each group were housed together in isolation from other cattle in an outdoor group pen with a covering to provide shelter from sun and rain. Calves in both groups were fed the same diet (calf grower ration and coastal Bermudagrass hay).

**Table 1:** Dairy calves and the total number of Baseline or Infected blood samples collected after pathogenic challenge.

Challenge	Calf ID	Age (months)	Weight (kg)	Baseline samples	Infected samples	Total
BRSV	1	3.2	135	4/7	4/4	8/11
	2	3.2	135	4/7	4/4	8/11
	3	3.0	130	4/7	4/4	8/11
	4	2.9	125	4/7	4/4	8/11
	5	2.4	125	4/7	4/5	8/12
*M. haemolytica*	6	6	204	3/4	3/3	6/7
	7	6	196	3/4	3/14	6/18
	8	6	145	3/4	3/3	6/7
	9	5	124	3/4	3/3	6/7
	10	5	142	3/4	3/3	6/7
Total samples				35/55	35/47	70/102

A subset of samples was selected for NMR metabolomic profiling because an even number of samples per category (Baseline or Infected) was needed to ensure variance and weight homogeneity of each data set for the multivariate analyses.

These dairy calves were subjected to two controlled challenge studies, each with a different infectious agent. The first group of dairy calves, age 5.6 months old (n = 5), was challenged with *M. haemolytica* (serotype A1, isolate D153) via bronchoalveolar lavage catheterization during the summer of 2019. The second group of dairy calves, age 2.9 months old (n = 5), was challenged with 5 mL of BRSV (GA-1, P5) delivered by a nebulizer (DeVilbiss Pulmo-Neb) through a custom-made face mask during the fall of the same year. The detailed procedures of pathogen preparation are described in the supplementary information (Supplementary Methods S1 online). The experiments were approved and carried out following the Mississippi State University-Institutional Animal Care and Use Committee guidelines and regulations (IACUC-19-037) and reported in compliance with the ARRIVE (Animal Research: Reporting In Vivo Experiments) guidelines 2.0. Because we wished to prevent life threatening conditions of calves due to experimental challenge, following the challenge, any calf exhibiting severe depression, or marked respiratory effort was treated with dexamethasone (0.1 mg/kg intravenously). One calf in the BRSV group exhibited signs of suspected secondary bacterial pneumonia (diagnosed by two veterinary clinicians) and was also treated with florfenicol (40 mg/kg). Calves with severe depression or marked respiratory effort in the *M. haemolytica* group were treated once daily for five days with ceftiofur (2.2 mg/kg subcutaneously).

A suite of clinical data was collected for 27 days during the bacterial challenge and for 34 days during the viral challenge in order to follow progression of asymptomatic, mild and severe degrees of disease following experimental challenge^15–17,21^. An example of the data collection sheet is provided in the Supplementary material (Supplementary Figure S1) and includes visual and clinical data such as the rectal temperature, heart and respiratory rate, presence or absence of depressed attitude, appetite for food, spontaneous or induced cough, nasal discharge, ocular discharge, submandibular lymphadenopathy, difficult breathing (dyspnea), and abnormal lung sounds (crackles, wheezes, or large airway sounds)^35^. Severe lung disease (difficult breathing, abnormal lung sounds) was differentiated from upper respiratory tract disease (nasal or ocular discharge). Objective measures (such as rectal temperature and respiratory rate) were taken by student workers, while subjective measures (such as difficult breathing and abnormal lung sounds) were scored by one of two licensed veterinarians who assessed these signs in all calves in the study. At the beginning of the study, the two veterinarians examined several calves together to agree on the criteria by which difficult breathing and abnormal lung sounds were identified.

In general, blood samples and visual and clinical data were collected for four days prior to challenge, for 11 continuous days after the challenge, and then every other day from day 12 onward post-challenge. The viral and bacterial challenge studies yielded a total of 97 and 105 blood samples, respectively. In the *M. hemolytica* challenge, samples were classified as Baseline (pre-infection), Asymptomatic (clinically normal or mild upper respiratory response after infection), Infected (clinically abnormal after the infection), Treated (infected and treated with ceftiofur (450 mg subcutaneously administered for five days)), or Recovered (clinically normal after the infection and ceftiofur treatment). Biochemical changes in the plasma are expected due to the use of antibiotics provided when aggravated signs of the disease were observed (established by the veterinarian and the approved IACUC protocol) or during the recovery processes. To avoid the interference of these changes in the interpretation of the NMR metabolomic profiles for healthy and sick calves infected *with M. hemolytica,* only data from the samples designated as Baseline (n = 20) and Infected (n = 26) were used in the univariate and multivariate analyses (Table 1, Supplementary Table S1 online). Before the BRSV challenge, the animals were tested for the absence of maternally derived anti-BRSV antibodies by a serum neutralizing assay. In the BRSV challenge samples were classified as Baseline (pre-infection), Asymptomatic (clinically normal or mild upper respiratory response) Infected (clinically abnormal with severe lung disease indicators), or Recovered (returned to clinically normal state). One animal died on day 7 and for lifesaving response the remaining individuals were administered one dose of dexamethasone (0.1 mg/kg; IACUC 19-037). Data from the BRSV blood samples designated as Baseline (n = 35) and Infected (n = 21) were used in the univariate and multivariate analyses (Table 1, Supplementary Table S1 online).

### Blood acquisition

Blood samples (n = 202) were drawn via jugular venipuncture and immediately placed on ice in two collection tubes containing the anticoagulant EDTA (ethylenediaminetetraacetic acid). The first tube was centrifuged at 4000 rpm for 20 min to separate plasma, and duplicates of 1 mL were stored at − 80 °C until NMR analysis. The second tube was used for complete blood counts (CBC), where red blood cells (RBC), hematocrit (HTC), hemoglobin (HGB), white blood cells (WBC), and platelet (PTL) contents were acquired using a veterinary hematology analyzer (Advia 2120i hematology analyzer, Siemens Healthcare Diagnostics Inc., Tarrytown, NY, USA). In addition, microscopic differential counts of WBC were performed by one co-author (EB) under the supervision of one of the board-certified veterinary clinical pathologists in the College of Veterinary Medicine (CVM) Diagnostic Laboratory to assess the variability of neutrophils, eosinophils, basophils, monocytes, and lymphocytes.

### Preparation of plasma for ^1^H-NMR analysis

Before collecting NMR spectra, proteins and larger macromolecules were removed from the samples using filters with a 3K molecular weight cutoff (Microsep, Pall Corporation, Ann Arbor, MI). Following filtration, 330 µL of each filtered plasma sample was mixed with 330 µL of sterile referencing solution^36^, (200 mM sodium phosphate buffer, 1 mM 3-trimethylsilylpropionate 2, 2, 3, 3-d4 (TMSP-d4, Cambridge Isotope Labs DLM-48-5) and 0.1% (w/v) sodium azide (NaN_3_) in 50% deuterium oxide (D_2_O, Cambridge Isotope Labs DLM-4 99)). Six hundred μL of the mixture was transferred to a clean NMR tube (Wilmad LabGlass, 535-PP-7) and kept at 4 °C for less than 24 h before NMR data acquisition.

### ^1^H-NMR spectra collection

For each pathogen challenge, an equal number of plasma samples (n = 70) from the Baseline and Infected stages were chosen for NMR analysis to ensure homogeneity of the variance and weight of each data set for the statistical analyses (Table 1); the detailed information from these samples can be found in the supplementary material (Supplementary Table S1 online). A Bruker Avance III HD 500 MHz spectrometer outfitted with a 5 mm BBFO probe (Bruker, Massachusetts, USA) was used for NMR spectroscopy. Samples were run in automation mode with a SampleJet, with all samples refrigerated at 4 °C until just prior to loading. A perfect-echo WATERGATE sequence (PE-WATERGATE, parameter set ZGESGPPE) was applied to collect the data^37^. Data were collected at 298 K for 128 scans with a 1 s inter-scan delay and a 3 s per-scan acquisition time. The total acquisition time for each sample, including 3D-shimming, was about 15 min. Topspin 4.0.8 (Bruker, Massachusetts, USA) was used for spectral processing following the acquisition. All spectra were zero-filled to 128k points, and a 1 Hz line broadening was used. Automatic phasing and baseline correction were performed, ^1^H-NMR spectra were segmented into successive non-overlapping regions of 0.0001-ppm chemical shifts between 0.0 and 10.5 ppm, and the water region was truncated between 4.30 and 5.10 ppm. The processed spectra were then imported into the Chenomx NMR Suite 8.6 (Chenomx, Edmonton, Canada) to identify individual metabolites and their concentration using a reference library containing 338 metabolites for 500 MHz spectrometers. Because the Chenomx library was acquired using a NOESY-based 1D pulse sequence, the reported metabolite concentrations (mM) are likely to differ slightly from the actual concentrations. However, relative differences between samples will be preserved, and absolute concentration differences for the majority of compounds are expected^38^.

### Statistics

The mean and standard deviation (SD) for temperatures, pulse rates, and respiratory rates (TPR), and CBC values collected from the Infected stage of the BRSV challenge (n = 21), the *M. haemolytica* challenge (n = 26), as well as the *combined* Baseline data points from *both challenges* (n = 55) were calculated using univariate statistics. The mean concentration (mM) and SD were also calculated similar to other studies^19,25,39^. The metabolites related to the referencing solution, diet ingredients, or those only detected in less than two samples in each group (Baseline or Infected) were excluded from the analyses. An ANOVA and pairwise mean comparison (Baseline vs. BRSV, Baseline vs. MH, BRSV vs. MH) using Tukey–Kramer HSD (honestly significant difference) test with alpha = 0.05 were used to assess for significance in parameter response for TPR, CBC, and metabolite concentration. The results for these post-hoc tests were reported with connecting letters, where different letters indicate significant differences between the Baseline, BRSV, and MH categories (JMP^®^ 14.0 SAS Institute Inc., NC. USA). In addition, a database representing a general state of infection labeled as V + B (viral + bacterial) was composed of data collected from all Infected samples, regardless of pathogenic agent, and was evaluated by pairwise mean comparison (Baseline vs. Infected) using Student’s *t*-test with alpha = 0.05.

### Multivariate analysis (MVA)

The CBC, visual and clinical data, and the metabolite concentrations were subject to Principal Component Analysis (PCA) using a full cross-validation (leave one sample out) and algorithm-singular value decomposition (SVD). We obtained correlation loadings plots to determine the magnitude and direction of a particular constituent’s contribution (Influence) to the models created for the Baseline and Infected stages (Unscrambler^®^ v. 11, Aspen Technology Inc., Massachusetts, USA). The processed ^1^H-NMR spectra contained spectral peaks ranging from 0.5 to 9.0 ppm that were chosen for the chemometrics-based MVA; peaks from free EDTA at 3.2 ppm and Ca^2+^-EDTA at 3.6 ppm were removed prior to analysis^40,41^. Three balanced datasets of spectra were created: the first labeled as Infected (V + B; n = 70) was created to represent a general infection state by combining information from both studies; the second corresponded to the BRSV challenge (n = 40), and the third to the *M. haemolytica* challenge (n = 30). SIMCA software-omics skin v.15.0 (Umetrics AB, Ume, Sweden) was used to apply pattern recognition methods. ^1^H-NMR spectra were subjected to PCA, in which the scale data conversion with mean-center scaling reflects the total metabolic differences between the two groups (Baseline vs. Infected) as well as the degree of variability within each group^42^.

The ^1^H-NMR spectra of plasma were analyzed using orthogonal partial least-squares discriminant analysis (OPLS-DA) to classify samples from the Baseline and Infected stages simultaneously. The OPLS-DA models were built with t[1]P and t[2]O, which stand for the first principal component and the second orthogonal component, respectively^42^. The OPLS-DA models were used to maximize the covariance between the measured data (X variable, peak intensities in ^1^H-NMR spectra) and the response variable (Y variable, predictive classifications). The quality of each model was assessed using R^2^X, R^2^, R^2^Y, and Q^2^, where R^2^X denotes the degree of optimization of the analytical model, R^2^ symbolizes the coefficient of determination, R^2^Y denotes the percentage of variance explained by the model, and Q^2^ describes the model’s cumulative prediction^42,43^. In the OPLS-DA models, segmented cross-validation (7 segments) with a random split was used to estimate the optimal component number of each model to avoid over-fitting. In addition, the percentage of accuracy, sensitivity, and specificity were calculated using the number of correct and incorrect predictions from the confusion matrix to test the ability of each model to identify true positive (Infected) and true negative (Baseline) samples correctly^44–46^. Permutation analysis was used to validate and to assess the reliability of the OPLS-DA models by randomly designating the class labels (Baseline or Infected) to different samples; then the discriminant model was carried out again an ‘n’ number of times (permutations = 200) with the incorrect class labels expecting poor classification values in comparison to the original calibration^47^. In permutation analysis a model is considered reliable if R^2^ > 0.4 and the intercept Q^2^ < 0^42,43,47^.

## Results

### Clinical and hematological results

**Figure 1:**
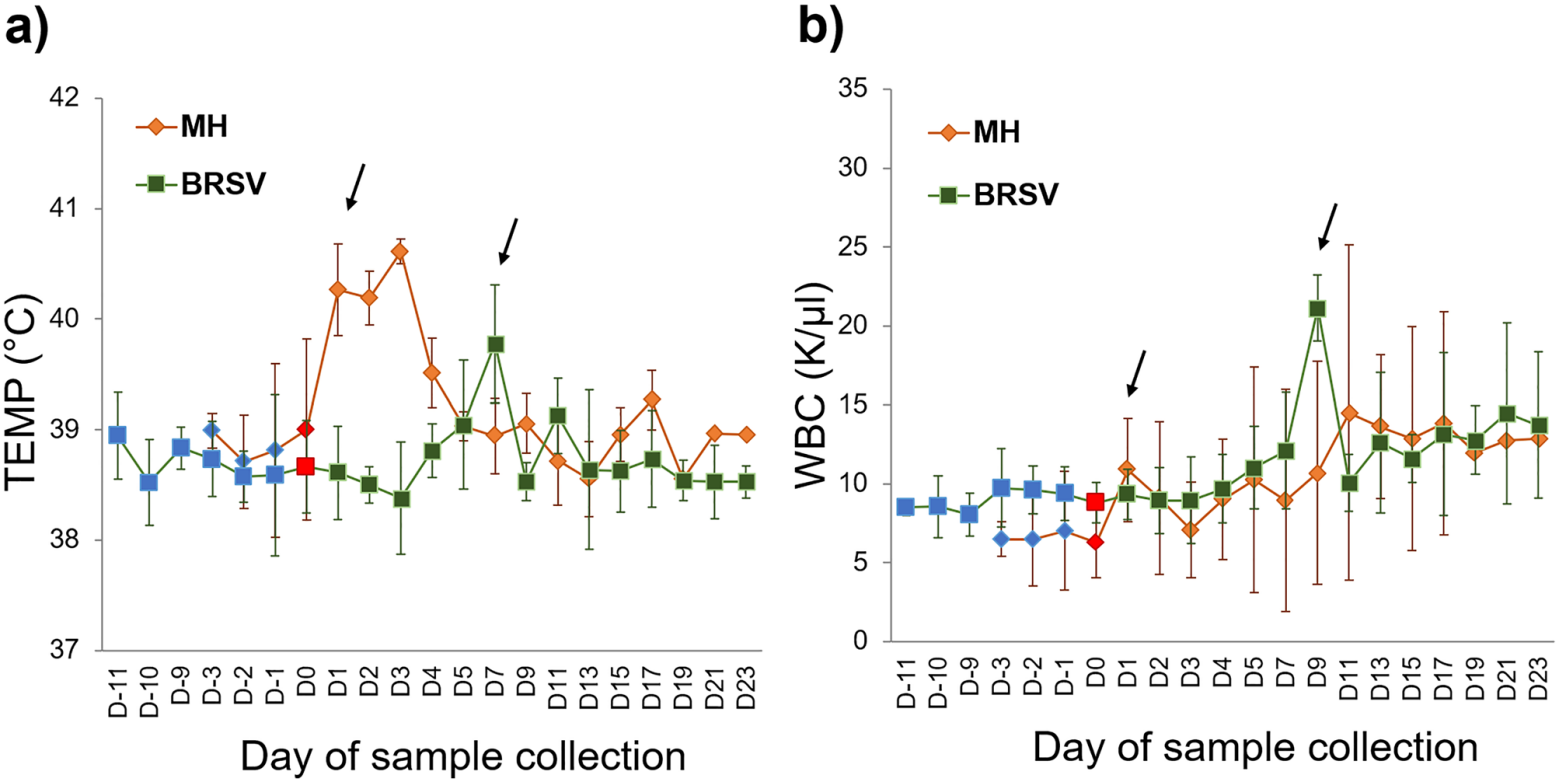
Dairy calves’ responses to viral (n = 5) and bacterial (n = 5) infection. Calves were challenged with the infectious agent on D0. (**a**) Daily rectal temperature (TEMP, °C) displayed as Mean ± SD. (**b**) WBC (thousands per cubic millimeter, K/µl) presented as Mean ± SD. A characteristic increase after signs of disease (indicated by arrows) in each challenge can be seen due to the activation of defense mechanisms against BRSV and *M. haemolytica* (MH) virulence factors. Markers in blue are the Baseline days; the red markers point to the day of the pathogenic challenge.

Overall, all the calves showed similar patterns of health and disease during the controlled studies (Fig. 1). The typical activation of the innate immunity or nonspecific defense mechanisms following the bacterial challenge were observed after 24 h of infection^48^ with an increase in rectal temperature (Fever) and WBC (Fig. 1a,b). Day 3 samples were collected before treating animals with ceftiofur as described above. The treatment could be associated with the recovery of the calves and the change in the temperature and WBC patterns (Fig. 1a,b). Mild signs of infection, including loss of appetite and nasal discharge from Day 1 until Day 19 were observed in calf 7; on D19, its signs intensified, presenting fever, depression, and increased purulent nasal discharge, and ceftiofur was administered as described above. By contrast, calves challenged with BRSV exhibited increased temperature and WBC on Day 7 post-infection (end of the asymptomatic phase), characteristic of the incubation period of this virus^8^. In the BRSV challenge on Day 7, calf 2 presented fever (≥ 40 °C), depression, difficulty breathing, and abnormal lung sounds. Blood samples were collected before providing dexamethasone (0.1 mg/kg) and the antibiotic florfenicol (40 mg/kg). Unfortunately, this calf died on Day 8. Given this calf’s death, dexamethasone was provided to the remaining calves on day 8 when they exhibited marked respiratory effort, as described above.

The results of the univariate and MVA tests for the TPR and CBC values for both challenges are shown in Table 2. In terms of the univariate analysis during the BRSV challenge, eight parameters were statistically different (*p* < 0.05) during Infection compared to the Baseline, where RBC, HCT, WBC, and the percentage of neutrophils were detected to increase while the percentage of lymphocytes, eosinophils, and basophils decreased. Throughout the *M. haemolytica* infection, four of the evaluated variables were statistically different (*p* < 0.05) from the Baseline values, with temperature, RR, and PLT increasing and HCT decreasing.

**Table 2:**
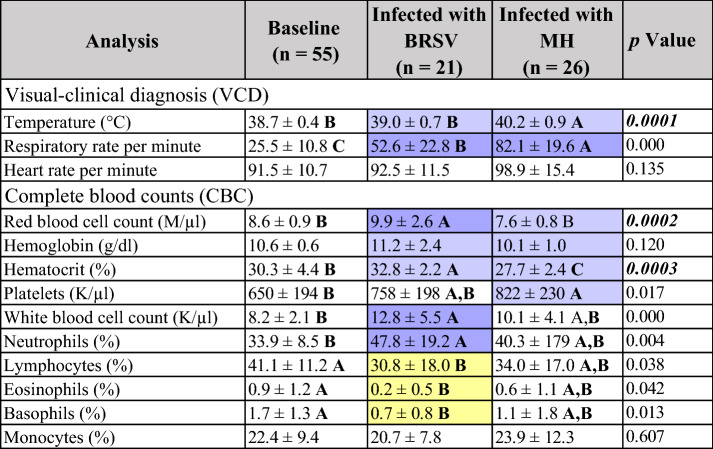
Clinical and hematological values (Mean ± SD) for dairy calves challenged with BRSV and *M. haemolytica* (MH).

Values with different letters were significantly different as determined by ANOVA (*p* < 0.05) between categories, with a significant increase (dark purple) or decrease (dark yellow) compared to the Baseline. Values positively correlated in the PCA during infection (light purple) compared with the Baseline. Italic = parameter changed in the ANOVA and the PCA, M/µl = Millions per microliter, g/dl = grams per deciliter, K/µl = thousands per cubic millimeter.

**Figure 2:**
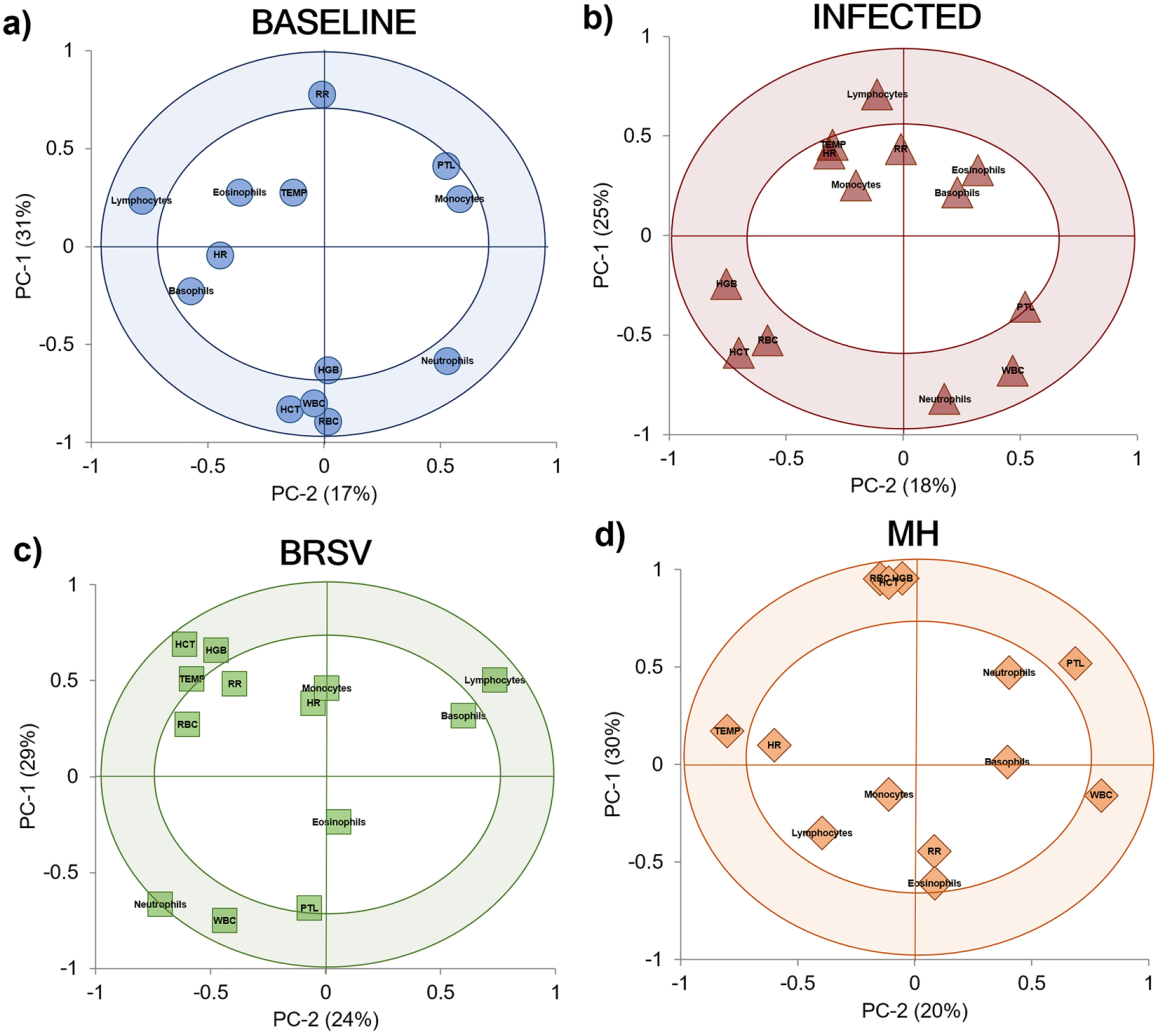
Principal component analysis (PCA) correlation loadings plots for TPR and CBC data. The variables inside the outer circle (colored area) have the most influence on database variability and are positively or negatively correlated within each model, while the parameters within the inner circle have low or no influence. (**a**) Baseline (n = 55); two PCs explained 48% of the variance. (**b**) General infection (V + B) database (n = 47) combined data from both studies, two PCs explained 43% of the variation of the database. (**c**) Infected with BRSV (n = 21), two PCs explained 53% of the variation of the database. (**d**) Infected with *M. haemolytica* (MH) (n = 26), two PCs explained 50% of the variation of the database.

The PCA correlation loading plots for TPR and CBC data (Fig. 2) show the relationships between the potential explanatory variables or evaluated parameters (X-matrix) within each of the databases (Baseline, Infected with virus or bacteria (V + B), BRSV, and MH) for the development of models^49^. The inner ellipse represents 50%, while the outer ellipse represents 100% of the explained variance for the individual variables. Thus, the area between the two ellipses explains 50–100% of the variance, implying that the centralized parameters (inside the inner circle) have an unimportant effect on the differentiation of each X-matrix. In contrast, those inside the outer circle (shaded) present a strong influence or significant impact on the differentiation of each model^49,50^. In addition, when the variables are placed in the positive or negative direction of the first principal component (PC-1), this influence can be described as positively or negatively correlated within each X- matrix, meaning the variables in those directions increase or decrease together to generate each model’s characteristic patterns^49,50^.

The distribution of TPR and CBC variables for the Baseline model can be seen in Fig. 2a; this pattern was compared by visual observation with those obtained in the Infected stage of each challenge separated and together. When evaluating the general infection database (Fig. 2b), similar patterns in the distribution of the evaluated parameters were observed in comparison to the Baseline, with the only difference observed in the position of HGB, which was negatively correlated during the infection and had no influence in the Baseline model. For the BRSV Infection model (Fig. 2c), TEMP, HGB, and HCT were positively correlated in comparison to the Baseline. The model for the evaluated variables during the *M. haemolytica* infection (Fig. 2d) differed from the Baseline in which temperature, HGB, HCT, and additionally RBC and PLT were positively correlated.

### Metabolites detected by ^1^H-NMR

**Figure 3:**
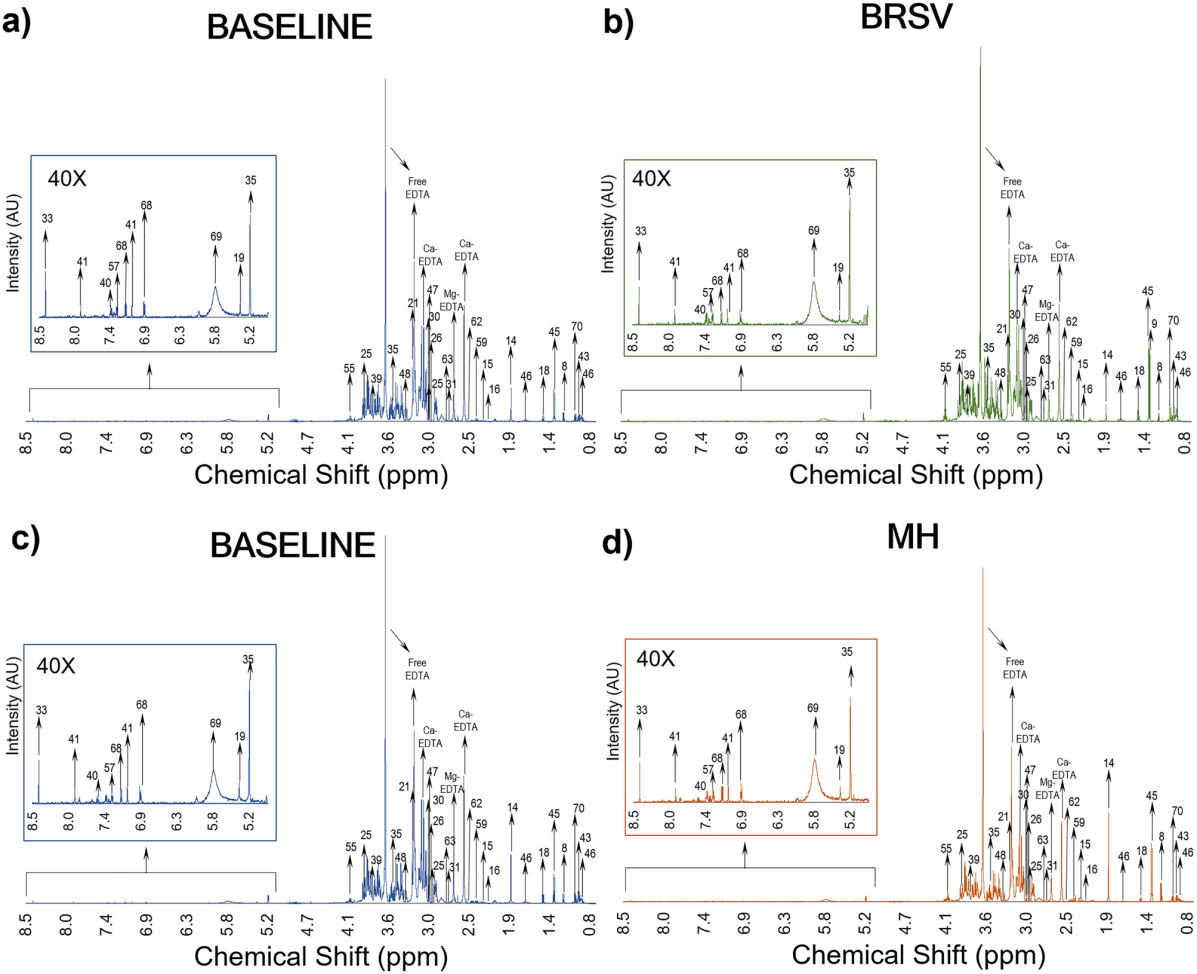
^1^H-NMR spectra (0.8–9.0 ppm) showing the peak intensities of metabolites present in plasma after the controlled infections with the main causal agents of BRD. (**a**) BRSV sample 66 (D0, calf 4), (**b**) BRSV sample 29 (D9, calf 4), (**c**) *M. haemolytica* (MH) sample 38 (D0, calf 6), (**d**) MH sample 2 (D2, calf 6). To improve the visualization of the peaks, the size of the region between 5.1–8.5 ppm was increased 40X.

The representative 500 MHz ^1^H-NMR spectra of plasma collected with EDTA from dairy calves before and after challenge are shown in Fig. 3. A total of 179 metabolites were identified using the batch profiler option in the Chenomx library. From these, a total of 72 metabolites were selected for the statistical analyses following the criteria explained in the methodology. After the selection, the presence of these metabolites was reliably identified in the samples manually using Chenomx. These compounds reproducibly appeared as well-resolved signals in the ^1^H-NMR spectra, and overlapped signals were confirmed using at least two peak groups before fitting in the Chenomx profiler. After the manual confirmation of these metabolites, a mean of 43 ± 8 of the selected metabolites were identified in each sample. The full identification and chemical classification of the 72 selected metabolites can be found in the supplementary material (Supplementary Table S2 online).

The findings from the univariate and multivariate analyses are shown in Tables 3 and 4 and include only the metabolites that presented a change during the Infection in comparison to the Baseline stage. The results for the general (V + B) infection database, which included data points from both viral and bacterial challenges, can be seen in Table 3. Regardless of the causative agent, 11 metabolites were found to change significantly (*p* < 0.05) compared to Baseline levels, where increases in 2-hydroxybutyrate and 3-hydroxyisobutyrate, and decreases in succinylacetone, isobutyrate, 2-hydroxyvalerate, acetone, O-acetylcholine, isoleucine, dimethyl sulfone, allantoin, and ethanol, were observed. Examining the response to specific pathogenic agents (Table 4) revealed the concentrations of a subset of six metabolites (BRSV = 2, MH = 4) changed significantly (*p* < 0.05) compared to the Baseline. Infection with BRSV resulted in a significant (*p* < 0.05) increase in guanidoacetate and a decrease in ethanol. On the other hand, infection with *M. haemolytica* showed significant (*p* < 0.05) increases in concentrations of 2-hydroxybutyrate, acetone, and 3-hydroxyisobutyrate, and a decrease in isobutyrate in comparison to the Baseline.

**Table 3:**
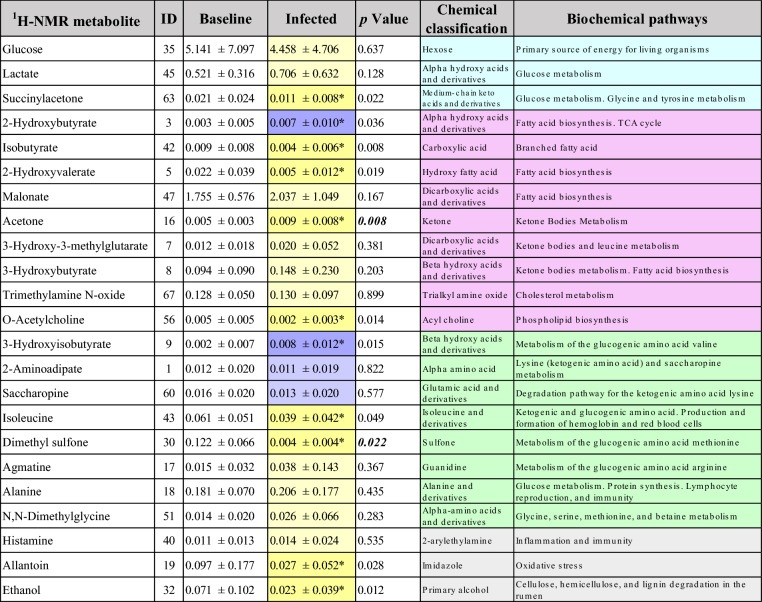
Baseline vs. Infected (V + B) changes in metabolite concentration.

Values are presented as mean ± SD. Data were analyzed using Student’s *t*-test with an α < 0.05 delineating significant treatment effects (*), increase (dark purple), decrease (dark yellow). Values positively (light purple) or negatively correlated (light yellow) in the PCA during infection compared with the Baseline. Biochemical pathways: Grey = other; light blue = glycolysis/gluconeogenesis pathways, magenta = triglycerides pathways, light green = protein pathways. Italic = parameter changed in both the ANOVA and the PCA.

**Table 4:**
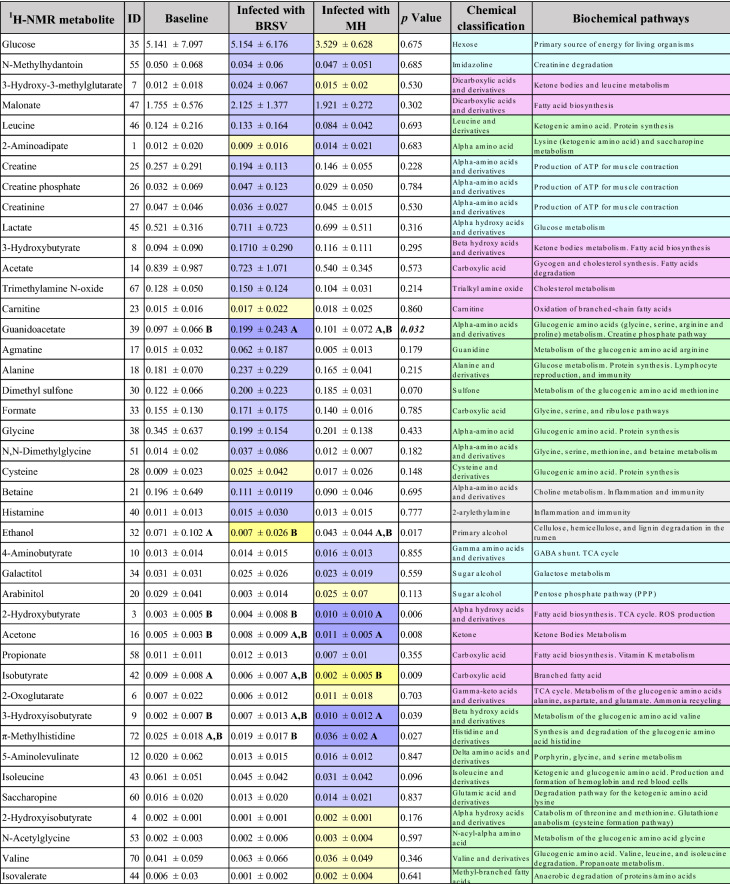
Metabolite concentration changes for plasma from dairy calves challenged with BRSV and *M. haemolytica*.

Values are presented as mean ± SD. Categories not connected by the same letter between Baseline, BRSV, and MH were significantly different (*p* < 0.05); increase (dark purple), decrease (dark yellow). Values positively (light purple) or negatively correlated (light yellow) in the PCA during infection compared with the Baseline. Biochemical pathways: Grey = other; light blue = glycolysis/gluconeogenesis pathways, magenta = triglycerides pathways, light green = protein pathways. Italic = parameter changed in the ANOVA and the PCA.

**Figure 4:**
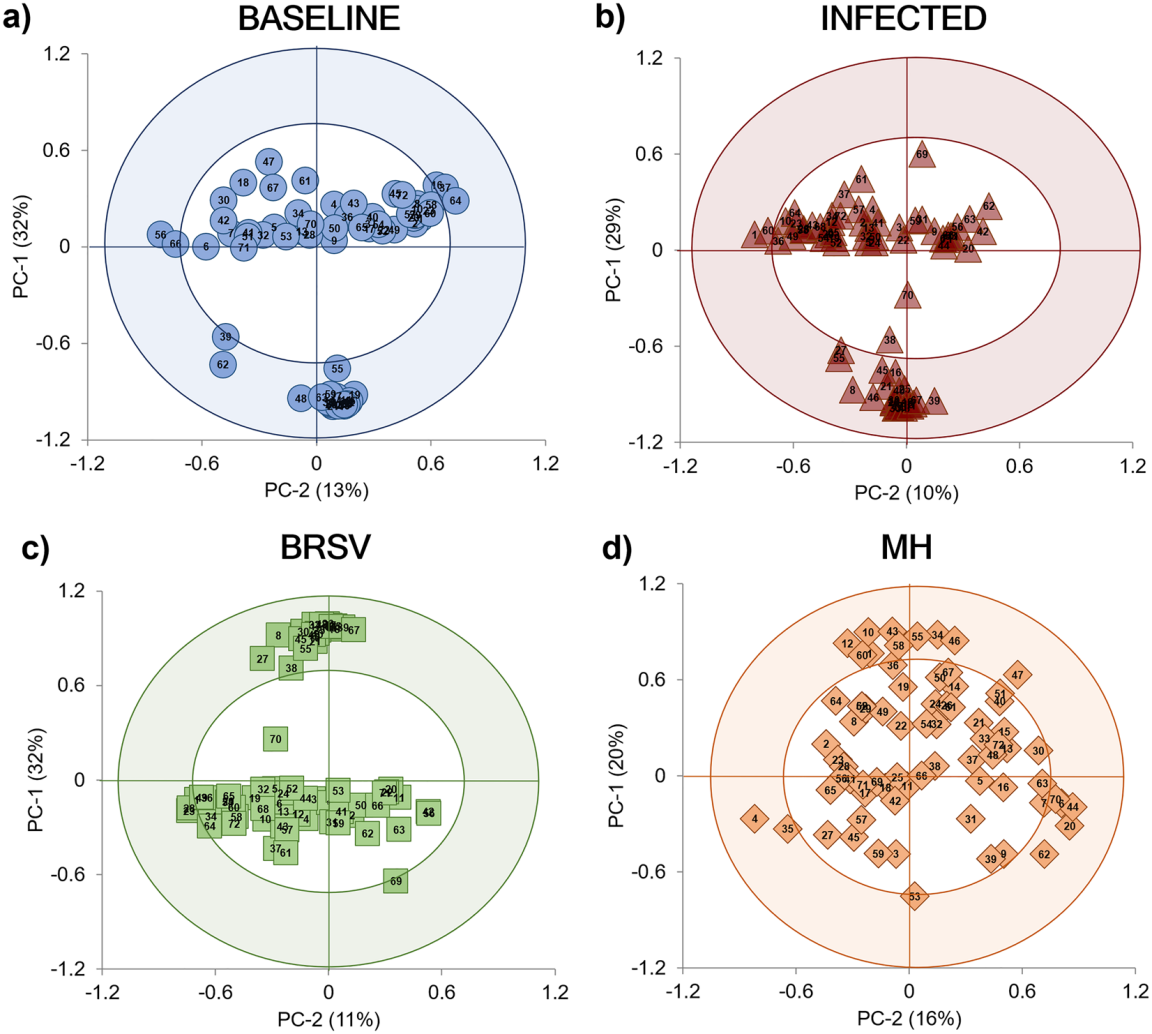
Principal component analysis (PCA) correlation loadings plots for the concentration of the selected ^1^H-NMR metabolites (n = 72) in plasma from dairy calves. The variables inside the outer circle (colored area) have the greatest influence on database variability and are positively or negatively correlated during the Baseline or Infected stages; the points inside the inner circle are thought to have low or no influence. (**a**) Baseline (n = 35); two PCs explained 45% of the variance. (**b**) Infected (V + B) data points from both challenge studies (n = 35), two PCs explained 39% of the variation of the database. (**c**) Infected calves with BRSV (n = 20), two PCs explained 43% of the variation of the database. (**d**) Infected calves with *M. haemolytica* (n = 15), two PCs explained 36% of the database variation. Each plasma sample, on average, contained 43 ± 8 of the selected metabolites.

The PCA correlation loading plots revealed distinct metabolites influencing the patterns for each model (Fig. 4). The distribution of metabolites differentiating the Baseline model (Fig. 4a) was compared by visual observation with the patterns from the three Infection models (Infected (V + B), BRSV, MH). In the model for the general V + B infection database (Fig. 4b), two metabolites (2-aminoadipate and saccharopine) were positively correlated, and 12 metabolites (glucose, lactate, malonate, acetone, 3-hydroxy-3-methylglutarate, 3-hydroxybutyrate, trimethylamine N-oxide, dimethyl sulfone, agmatine, alanine, N,N-dimethylglycine, and histamine) were negatively correlated when compared with the Baseline model. In the PCA model for each pathogen, 36 metabolites (BRSV = 25, MH = 23) changed in comparison to the Baseline model (Table 4). Of these 48 metabolites, glucose, N-methylhydantoin, 3-hydroxy-3-methylglutarate, malonate, leucine, and 2-aminoadipate changed in response to both pathogens (Table 4). In the PCA describing BRSV infection (Fig. 4c), 21 metabolites (glucose, N-methylhydantoin, 3-hydroxy-3-methylglutarate, malonate, leucine, creatine, creatine phosphate, creatinine, lactate, 3-hydroxybutyrate, acetate, trimethylamine N-oxide, guanidoacetate, agmatine, alanine, dimethyl sulfone, formate, glycine, ,N,N-dimethylglycine, betaine, and histamine) were positively correlated. Only three metabolites (2-Aminoadipate, carnitine and cysteine) were negatively correlated in the BRSV infection in comparison to the Baseline. By contrast, in the *M. haemolytica* infection (Fig. 4d), ten metabolites were positively correlated (N-methylhydantoin, malonate, leucine, 2-aminoadipate, 4-aminobutyrate, galactitol, propionate, 5-aminolevulinate, isoleucine, and saccharopine), and eight metabolites were negatively correlated (glucose, 3-hydroxy-3-methylglutarate, arabinitol, 2-oxoglutarate, 2-hydroxyisobutyrate, N-acetylglycine, valine, and isovalerate).

### ^1^H-NMR Chemometrics-based multivariate analysis

**Figure 5:**
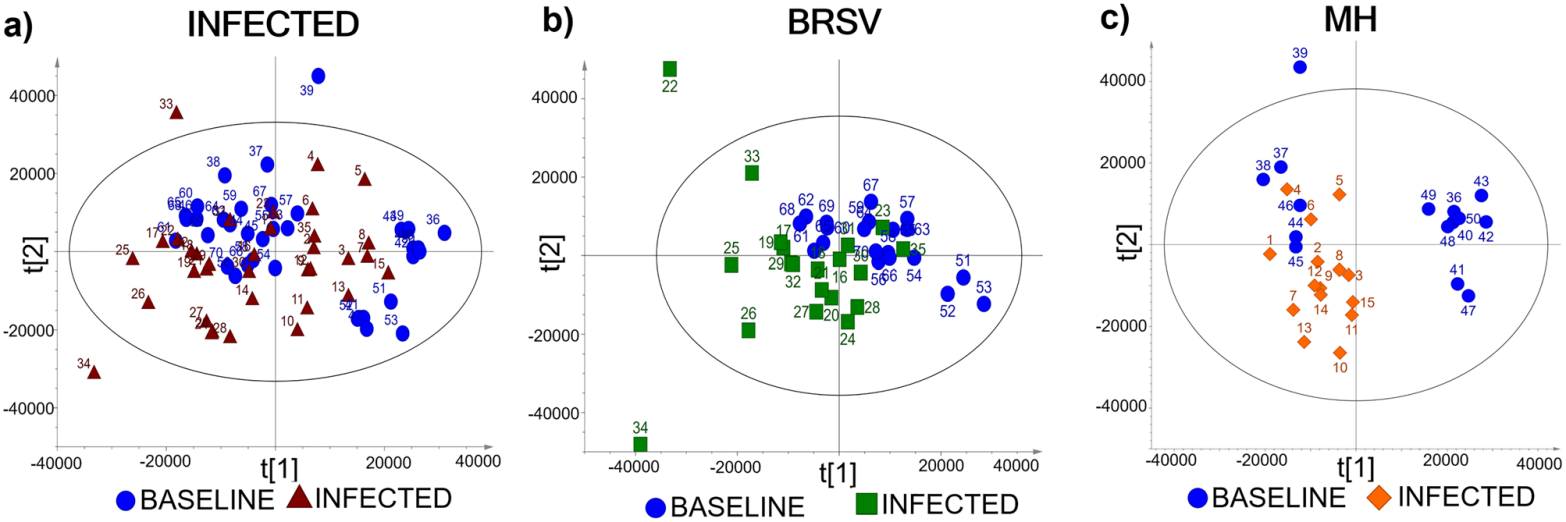
Principal component analysis (PCA) scores plots for ^1^H-NMR spectra from Baseline and Infected plasma samples. (**a**) PCA scores plot (n = 70) from the combined V + B infection database (PC-2: R^2^X = 0.34, Q^2^ = 0.26). (**b**) PCA scores plot (n = 40) from the BRSV challenge (PC-2: R^2^X = 0.34, Q^2^ = 0.10). (**c**) PCA scores plot (n = 30) from the *M. haemolytica* (MH) challenge (PC-2: R^2^X = 0.45, Q^2^ = 0.25). Labels above the scores indicate the sample ID (Table S1).

The PCA scores plots showing the trends of the ^1^H-NMR spectral signals can be seen in Fig. 5. In the scores plot, each point corresponds to either a Baseline or Infected sample, and overlap between the two groups indicates similarities in the metabolite composition of the plasma. Outliers are samples outside the confidence ellipse based on Hotelling's T^2^ (significance level 0.05). The database for the general V + B infection was analyzed with 10 principal components (Fig. 5a), giving modeling parameters R^2^X = 0.83 and Q^2^ = 0.56; in this case, three outliers were identified, which corresponded to samples 33 (D9, calf 5), 34 (D11, calf 5), and 39 (D-2, calf 7). In the database of BRSV samples (Fig. 5b), seven principal components gave a degree of optimization R^2^X = 0.80 and a cumulative prediction Q^2^ = 0.38, with two outliers: samples 22 (D4, calf 2) and 34 (D11, calf 5). In the database composed of *M. haemolytica* samples (Fig. 5c), six principal components produced modeling parameters R^2^X = 0.79 and a Q^2^ = 0.48. The outlier corresponded to Baseline sample 39 (D-2, calf 7).

**Figure 6:**
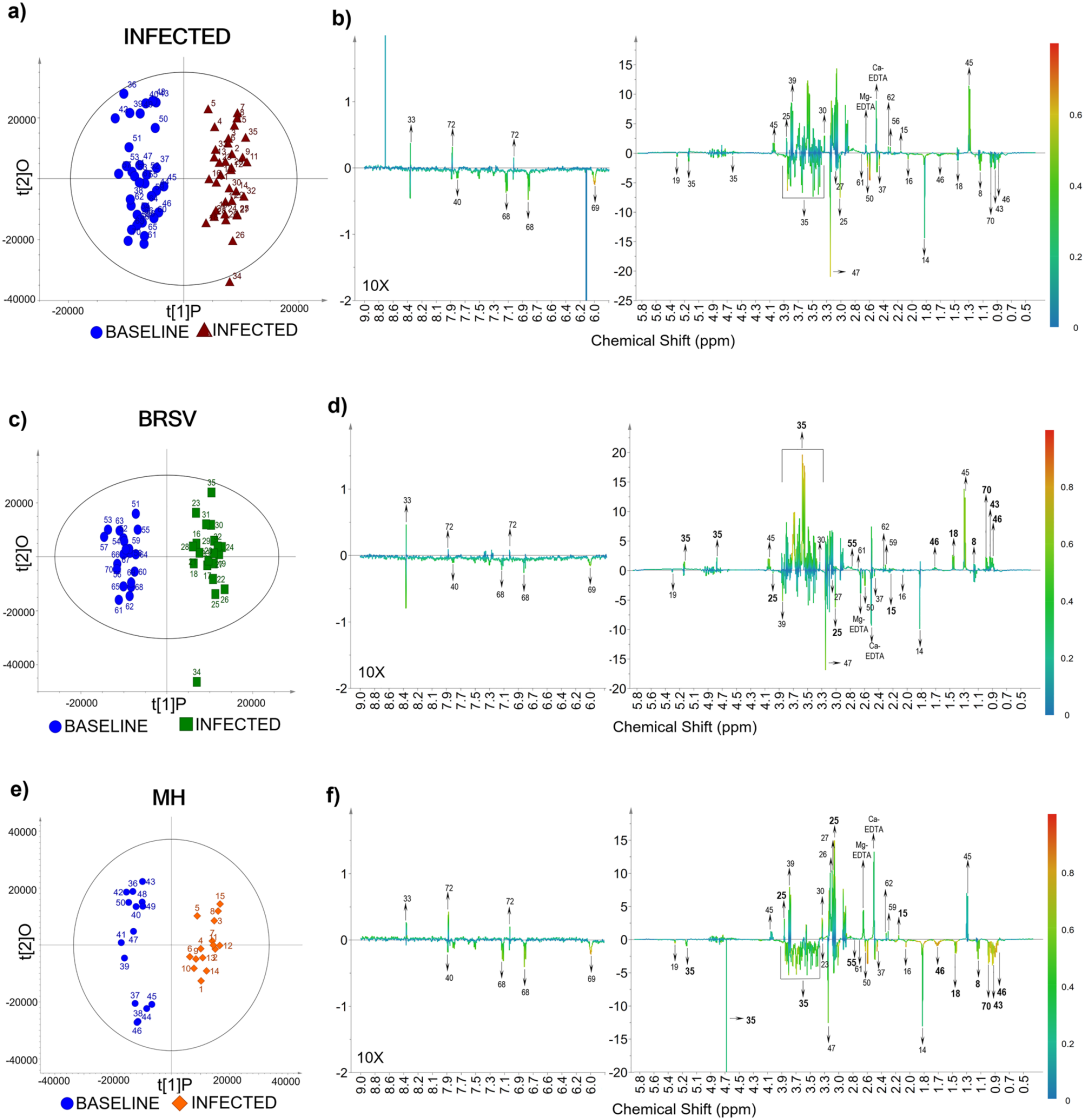
OPLS-DA scores plots resulting from ^1^H-NMR spectra of plasma as well as the corresponding coefficient loading plots. The color map depicts the significance of spectral signals between the two categories (Baseline and Infected). (**a**) OPLS-DA scores plot from the combined V + B infection database (n = 70). (**b**) Coefficient loadings plot for general infection. (**c**) OPLS-DA scores plot from the BRSV challenge (n = 40). (**d**) Coefficient loadings plot from the BRSV challenge. (**e**) OPLS-DA scores plot from the *M. haemolytica* (MH) challenge (n = 30). (**f**) Coefficient loadings plot from the *M. haemolytica* challenge. Labels above the scores indicate the sample’s ID, and above the peaks show the metabolite’s ID. To improve the visualization of the peaks in the coefficient loadings plots, the size of the region between 6.0–9.0 ppm was increased 10X.

The OPLS-DA scores plots (Fig. 6) for the general (V + B) infection (Fig. 6a), BRSV (Fig. 6c), and *M. haemolytica* (Fig. 6e) databases demonstrated a clear distinction in the chemistry of plasma from non-infected and infected cattle. This suggests that even though the chemical composition of the plasma is similar before and after infection, as shown in the PCA scores plots (Fig. 5), there is enough information to successfully detect and discriminate infection from baseline with an accuracy, sensitivity, and specificity higher than 95% (Table 5). More importantly, all the models showed a specificity of 100%, meaning no false-positive samples (Baseline samples) were classified as infected. Table 5 shows the quality parameters for the calibrations and validations of each model using only two principal components (PCs). The values of R^2^X (**> **0.4), R^2^ (**> **0.9), R^2^Y (**> **0.9), and Q^2^ (**> **0.4) obtained in the calibration indicated that the models are robust, reliable, and have a low risk of overfitting^42,51^. In addition, R^2^ values were greater than Q^2^ in the permutation plots, such that a more positive slope in the regression line corresponds to a higher degree of fit to the data and the reliability of each model^42,43,47^. The distance to model plots and permutation plots for the discriminant models can be found in the Supplementary material files (Supplementary Figure S1 online).

**Table 5:** OPLS-DA model quality parameters for the classification of ^1^H-NMR spectra from plasma collected before and after the controlled infections.

OPLS-DA MODELS	Baseline versus infected (V + B)	Baseline versus BRSV	Baseline versus MH
n	70	40	30
Cal R^2^X	0.67	0.39	0.45
Cal R^2^	0.94	0.96	0.94
Cal R^2^Y	1.00	1.00	1.00
Cal Q^2^	0.65	0.81	0.75
Accuracy (%)	98.6	97.5	100
Sensitivity (%)	97.1	95	100
Specificity (%)	100	100	100
Val R^2^	0.73	0.72	0.69
Val Q^2^	− 0.97	− 1.1	− 0.73
Metabolites contributing to the trends in the scores describing Baseline samples (t[1]P < 0)	3-Hydroxybutyrate, acetate, acetone, alanine, allantoin, glucose, glutamine, histamine, isoleucine, leucine, malonate, methylamine, sarcosine, tyrosine, urea, valine	Acetate, acetoacetate, acetone, allantoin, creatine, creatinine, glutamine, guanidoacetate, histamine, malonate, methylamine, tyrosine, urea	3-Hydroxybutyrate, acetate, acetone, alanine, allantoin, betaine, glucose, glutamine, histamine, isoleucine, leucine, malonate, methylamine, N-methylhydantoin, sarcosine, tyrosine, urea, valine
Metabolites contributing to the trends in the scores describing Infected samples (t[1]P > 0)	Acetoacetate, creatine, dimethyl sulfone, formate, guanidoacetate, lactate, pyruvate, succinate, π-methylhistidine	3-Hydroxybutyrate, alanine, dimethyl sulfone, formate, glucose, isoleucine, lactate, leucine, N-methylhydantoin, pyruvate, sarcosine, succinate, valine, π-methylhistidine	Acetoacetate, creatine, creatine phosphate, creatinine, dimethyl sulfone, formate, guanidoacetate, lactate, pyruvate, succinate, π-methylhistidine

Cal = calibration, Val = validation, R^2^X = degree of optimization, R^2^ = coefficient of determination, R^2^Y = percentage of variation explained by the model, and Q^2^ = cumulative prediction, t[1]P = first principal component.

The color map derived from the coefficient loading plot indicates significant changes in spectral signals, which contribute to the trends in the first principal component (t[1]P) of the OPLS-DA scores plot (Fig. 6) that distinguish Baseline from Infected samples. These spectral signals can be compared to known metabolite peaks, allowing them to be assigned in the loading plot. In Fig. 6, the peaks with positive values in the ^1^H-NMR coefficient loading plot contribute to the trends in the scores describing Infected samples (t[1]P > 0), whereas metabolites with negative peaks contribute to the trends in the scores describing Baseline samples (t[1]P < 0). Overall, similar trends in the coefficient loadings plots were observed for the general (V + B) infection database and the specific infection with *M. haemolytica,* and associated with the metabolites acetoacetate (ID = 15) and creatine. However, in the BRSV infection, these two metabolites were distinguishing contributions to Baseline classification (Table 5). In contrast, scores associated with BRSV infection have contributions from 3-hydroxybutyrate, alanine, glucose, isoleucine, leucine, N-methylhydantoin, and valine. Yet, these same metabolites contribute to the scores for the Baseline samples from the *M. haemolytica* and the general Infection (Table 5).

## Discussion

Induced experimental infections provide a more accurate data set because samples used in this analysis derive from animals that are actually infected; thus, metabolite information is correlated with clinical physiological signs and hematological parameters recorded before and after the controlled infections. In this study, ^1^H-NMR was used to determine the metabolomics of BRD by detecting biomarkers in plasma after dairy calves were challenged with two of the main causal agents of this disease under controlled conditions. The existing plasma NMR-BRD metabolome, which was acquired from dairy (1–2 month-old)^33^ and beef (1–2 years-old)^34^ cattle in non-controlled field conditions, used VCD as the reference method to determine the infection. However, the lack of identification of specific causal agents restricted the discovery of specialized biomarkers that might be targeted for the development, understanding, and validation of novel BRD diagnostic strategies such as NIRS-based detection in the early stages of the disease. As a result, the current study adds to, and expands on, the existing bovine metabolome by identifying distinctive biomarkers in dairy cattle infected with BRSV and *M. haemolytica* independently.

In general, viral and bacterial agents activate innate immunity, which is comprised of nonspecific defense mechanisms that are triggered shortly after the appearance of the antigen^52^. This occurs as a result of the production of a motif of molecules expressed by the pathogen known as pathogen-associated molecular patterns (PAMPs)^53,54^. In the case of BRSV, the PAMPs are known components consisting of glycoprotein G, fusion protein F, and single-stranded RNA^8^. For *M. haemolytica*, PAMPs include flagellin, lipopolysaccharide (LPS) complex, and leukotoxin (LKT)^55^. These elicitors are identified by pattern recognition receptors (PRRs) for rapid detection of the threat from a potential pathogen^53^. Surface-bound and intracellular PRRs, such as Toll-like receptors (TLRs), nucleotide-binding and oligomerization domain (NOD)-like receptors, and RNA helicases, are expressed by bovine respiratory tract cells^54^. The engagement of PAMPs by PRRs results in the production of damage-associated molecular patterns (DAMPs), initiating ATP-dependent signaling cascades. Activated transcription factors induce the production of inflammatory cytokines and chemokines for release into the body, which attracts neutrophils, macrophages, and lymphocytes to the respiratory tract, resulting in respiratory disease^7,8,53^.

Ruminants have a specialized digestive system to degrade grass and get the necessary nutrients to maintain homeostasis, and most of the glucose comes from the gluconeogenesis of oxaloacetate obtained from the propionate produced by the microorganism species *Megasphaera*, *Veillonella,* and *Selenomonas* in the rumen^56,57^. Because this is a slow process, supplying the energy demands during the cell signaling cascades and immune response caused by PAMPs recognition requires metabolism reprogramming by immune cells, where alternative energy sources such as triglycerides and proteins are used for ATP production^58–60^. Glucose is an upstream regulator of 26 genes associated with BRD in dairy cattle, and if glucose homeostasis is disrupted, hypoglycemia or hyperglycemia occurs^61^. The negative correlation of glucose in the PCA model observed in the present *M. haemolytica* challenge and in the combined V + B database is in line with the decreases of this metabolite reported in BRD studies in dairy and beef cattle as a result of natural or artificially induced infections, LPS injections, stress-related to transport, and receiving calves^34,61,62^. It has been suggested that, in addition to the metabolic changes caused by the immunological response, the decrease in glucose levels is also due to the hypoglycemic effect of BRD and the decline in diet due to the discomfort caused by the respiratory signs^34^. In contrast, a positive correlation in glucose was observed in response to BRSV infection. However, it is likely that the differential change in glucose arose as a response to the life-saving administration of the glucocorticoid drug dexamethasone to calves in the BRSV challenge, and is a limitation of the study.

To meet the energy demands due to the recruitment of inflammatory cells and the phagocytotic processes for microbial death, immune cells such as neutrophils, monocytes, macrophages, and lymphocytes undergo aerobic glycolysis^59,60^. In this scenario, pyruvate does not enter the mitochondrion but is instead metabolized to lactate in the cytoplasm, with glycolysis rapidly providing minor amounts of ATP^58,59^. A positive correlation of lactate was observed in the PCA for the BRSV challenge. Similarly, in combination challenges with BHV-1 and *M. heamolytica* carried out in beef cattle, animals that died had higher lactate concentrations than those that survived^63^. It has been reported that the decrease in oxygen levels during BRD due to stress, blockage of the respiratory tract with mucous secretions, and lung inflammation also increased lactate concentration, the likelihood of disease progression, and eventual mortality in dairy cattle^64^. Aerobic metabolism in neutrophils is associated with an increase in reactive oxygen species (ROS), an important source of bactericidal activity^59^. A significant increase in 2-hydroxybutyrate, which is a metabolite associated with ROS production and lipid oxidation (ß-oxidation), was detectedin the present study in the *M. haemolytica* challenge and had also previously been detected in rumen fluid, serum, milk, and in greater amounts in the urine of six lactating and six non-lactating Holstein dairy cows, as well as in the mammary gland of the lactating cows^65^.

To offset the consequences of metabolic diseases involving energy imbalance, ruminants and, more specifically, bovine species are known to use the alternative triglyceride route^27–30,32^. Triglycerides are made up of glycerol and fatty acids. Fatty acids undergo ß-oxidation in the liver, producing acetyl-CoA, which enters the tricarboxylic acid (TCA) cycle. As a result, the reducing agents NADH and FADH_2_ are produced, which feed the electron transport chain and drive large amounts of ATP production to address energy imbalance^59,66^. Large amounts of acetyl-CoA exceeding the capacity of the TCA cycle result in the generation of ketone bodies^58,66^. During BRD in dairy cattle, glucose and oxygen uptake are reduced, resulting in increased ketone body formation^67,68^. Under such conditions, the metabolites 3-hydroxybutyrate, acetoacetate, and acetone accumulate downstream of acetyl-CoA generation^67,69^. A positive PCA correlation in 3-hydroxybutyrate as detected here in the BRSV challenge is consistent with previously reported increases in this metabolite as being important for differentiating healthy from sick cattle with BRD^34^ and having a negative effect on neutrophil function and recruitment, consequently allowing pneumonia to progress^70^. The plasma collected during the *M. haemolytica* challenge presented significant increases or an association with the Infected stage in the discriminant analysis in the ketone bodies acetone and acetoacetate, respectively. Moreover, the dicarboxylic acids malonate and 3-hydroxy-3-methylglutarate were detected in samples from both challenge studies. Taken together, these results support the idea that higher energy supplies derived from fatty acid pathways could be required to counteract the energy imbalance caused by the immune response to the secondary bacterial infection rather than the initial viral infection, even if glucocorticoids are administered.

The alternative protein pathway is the final resource used to overcome increased metabolic demands^71^. Changes in the concentrations of glucogenic amino acids, ketogenic amino acids, and their metabolites were detected during both challenge, similar to previous studies in cattle with ketosis caused by negative energy balance^72^. Here, a negative PCA correlation of the alpha-amino acid 2-aminoadipate was detected in response to the BRSV challenge; contrastingly, in the *M. haemolytica* challenge, a positive correlation was found. This metabolite is an intermediate in the metabolism of the ketogenic amino acid lysine^25^. During the BRSV challenge, positive correlations in the PCA for the concentration of the alpha-amino acids creatine, creatine phosphate, and creatinine, and the imidazoline N-methylhydantoin suggested that BRSV could be causing viral myositis, affecting muscle health by increasing the metabolites involved in the regeneration of ATP in skeletal muscle to energize muscle contraction^73^. In the controlled infection with *M. haemolytica*, the glucogenic amino acid valine was negatively correlated in the PCA, while isoleucine which can be glucogenic or ketogenic, presented a positive correlation. In contrast, previous research demonstrated that injecting LPS from M*. haemolytica* into feed cattle reduced the levels of isoleucine in plasma while increasing alanine^74,75^.

In this work, chemometric-based MVA successfully distinguished the ^1^H-NMR spectra from bovine plasma collected during the Baseline and Infected stages of both challenge studies with an accuracy, sensitivity, and specificity ≥ 95%. Importantly, the successful group differentiation suggests that, while there were some differences in the age, weight, collection season, and physiological response to infection by some of the dairy calves between the two challenge studies, these did not interfere with the chemical content of plasma required for the classification of the samples using this analytical technique; thus, the metabolomic profile presented here is reliable and can be added to the current BRD metabolome as new information to understand the biochemical pathways involved in this disease. In addition, these findings indicate biochemical differences between healthy and sick animals with two of the main causal agents of BRD, where metabolites related to homeostasis in the baseline and energy imbalance during the infections were found to influence the discrimination plots. Previous research using NIRS and NMR also successfully discriminated plasma from dairy and beef cattle with BRD, with sensitivities and specificities close to 90% when using clinical diagnosis as the reference method^15,33,34^. The findings in the current study are also consistent with previous research that used discriminant analysis on NMR spectra of plasma from dairy cattle to identify the metabolomics of animals with ketosis, ovarian quiescence, and fatty liver disorder^27,29–32^ showing the potential of this technique for the detection of metabolic disorders related with nutrition, reproduction, and disease.

## Conclusion

By using ^1^H-NMR spectroscopy in blood plasma, this study demonstrated that important metabolic shifts are occurring in the host in response to infection with BRSV or *M. haemolytica*. Following the application of univariate and multivariate statistical methods, the concentration of 46 metabolites (BRSV = 32, MH = 33) changed in comparison to the Baseline stage. These metabolites appeared to be critical fuel substrates and products of the energy imbalance occurring during the infections due to signaling cascades and immune response activation. In addition, our findings support the potential of NMR to create metabolic profiles of BRD that contribute to the understanding of the diversity and concentrations of essential metabolites in plasma that can be applied for the further development of novel diagnostic tools.

## Supplementary Information

Supplementary Information.

## Data Availability

Data is available upon request from Carrie K. Vance (ckv7@msstate.edu).
