# Supplementary material for: NMR-based metabolomics of plasma from dairy calves infected with two primary causal agents of bovine respiratory disease (BRD)
Source: Sci Rep. 2023 Feb 15;13:2671. doi: 10.1038/s41598-023-29234-3 (PMC9930073; doi:10.1038/s41598-023-29234-3)
Supplement: Supplementary file 1 — Supplementary Information. [file 41598_2023_29234_MOESM1_ESM.pdf]

NMR-based metabolomics of plasma from dairy calves infected with two primary causal agents of Bovine Respiratory Disease (BRD)

Mariana Santos-Rivera[1], Nicholas C. Fitzkee[2], Rebecca A. Hill[2], Richard E. Baird[1], Ellianna Blair[1], Merrilee Thoresen[3], Amelia R. Woolums[3], Florencia Meyer[1], and Carrie K. Vance[1]

[1] Mississippi State University, Department of Biochemistry, Molecular Biology, Entomology, and Plant Pathology, Mississippi State, MS, USA 39762.
[2] Mississippi State University, Department of Chemistry, Mississippi State, MS, USA 39762[3] Mississippi State
[3] University, College of Veterinary Medicine, Pathobiology & Population Medicine, Mississippi State, MS, USA 39762

Supplementary Methods S1. Pathogen preparation and challenge administration

Bacterial Challenge

The authors previously described this protocol in a recent publication[1]. In preparation for the controlled bacterial challenge, *M. haemolytica* isolate D153 was streaked onto BD Brain Heart Infusion (BHI) agar (BactoTM 237500) then incubated at 37 °C overnight. For the starter culture, a single colony was selected and inoculated in 5 mL BHI broth (DifcoTM 241830) and incubated overnight at 200 rpm at 37 °C in a shaker incubator, then diluted 1:100 and incubated again overnight. On the day of the challenge study, the culture was diluted again 1:100 and centrifugated at 200 rpm and 37 °C until Abs_{600} = 1.0, then bacteria were pelleted at 10,000 rpm for 10 minutes at 4 °C and then resuspended to a final concentration of 1.0 x 10^9cfu/30 mL in 0.9 % saline. The challenge inoculum was administered at 1.0 x 10^9 cfu for every 91 kg of calf body weight. The bacterial challenge was given via bronchoalveolar lavage (BAL) catheter. Briefly, each calf was physically restrained in a chute, and a halter and lead were used to position the calf's head straight up. Five mL of 2 % lidocaine was used as a local anesthetic and was squirted into one nostril, and the BAL tube was introduced through this nostril and gently advanced into the trachea until the end of the catheter wedged into a bronchus. The appropriate volume of challenge inoculum, based on body weight, was administered through the BAL catheter, followed immediately by 60 mL of sterile 0.9 % saline and 120 mL of air. Following the challenge procedure, a sub-sample of the challenge inoculum was used to prepare a quantitative culture assay on BHI agar and was checked at 24 - 48 h, and cfu were counted at 96 - 120 h.

Viral Challenge

Part of this protocol was described in a recent publication by the authors[2]. Seven days prior to challenge MDBK (Madin-Darby bovine kidney), cells were seeded at a density of 40,000 cells / cm^2 into T-75 tissue culture flasks containing DMEM amended with 10 % fetal bovine serum and 2 mM Glutamax (Gibco). The cultures were maintained at 37 °C at 5 % $CO_{2,}$ and cells were given approximately 4-6 h to adhere prior to infecting them with BRSV (GA-1, P5). To infect the cells, media was aspirated, and the cell monolayer in each flask was gently rinsed with 10 mL of PBS. Following removal of the PBS, 1 mL of virus suspension was added to each of the flasks, and 1 mL of complete cell culture media was added to an additional flask to provide an infection negative control. Flasks were gently rocked to ensure that the 1 mL of virus suspension or media alone covered the entire cell culture surface and were returned to the incubator for 30 minutes. After 30 minutes, 12 mL of fresh pre-warmed media was added to each flask and then returned to the incubator, where they remained in culture for 7 days. Cell monolayers were monitored visually at 4X daily from 72 h post-infection up to the day of challenge to track the formation and propagation of cytopathogenic effect (CPE) in the infected flasks. On the day of the challenge, the media from the BRSV infected flasks was pooled into a sterile 50 mL tube, mixed well and five 5 mL aliquots were prepared (1/calf). For the determination of viral titer, 24-well plates were seeded with MDBK cells utilizing the same technique described for the culture flasks. Using a serial dilution technique, 3 wells were treated with 10^{-1} of viral suspension, and serial dilutions from these wells were carried out to 10^{-15}. This was done for virus suspension collected prior to the challenge and was repeated with another aliquot of the virus that was exposed to the same conditions as the challenge aliquots. TCID50 plates were monitored for 13 days until no further change was observed, and the viral titer was calculated and determined to be TCID50 -5.20 for both pre-and post-challenge samples. For the challenge, 5 mL of BRSV (GA-1, P5) was administered via nebulizer (DeVilbiss Pulmo-Neb) through a custom-made face mask. The challenge procedure took approximately 20 - 25 minutes per calf.

Supplementary Figure S1. Visual and clinical data recording form for dairy calves with BRD.

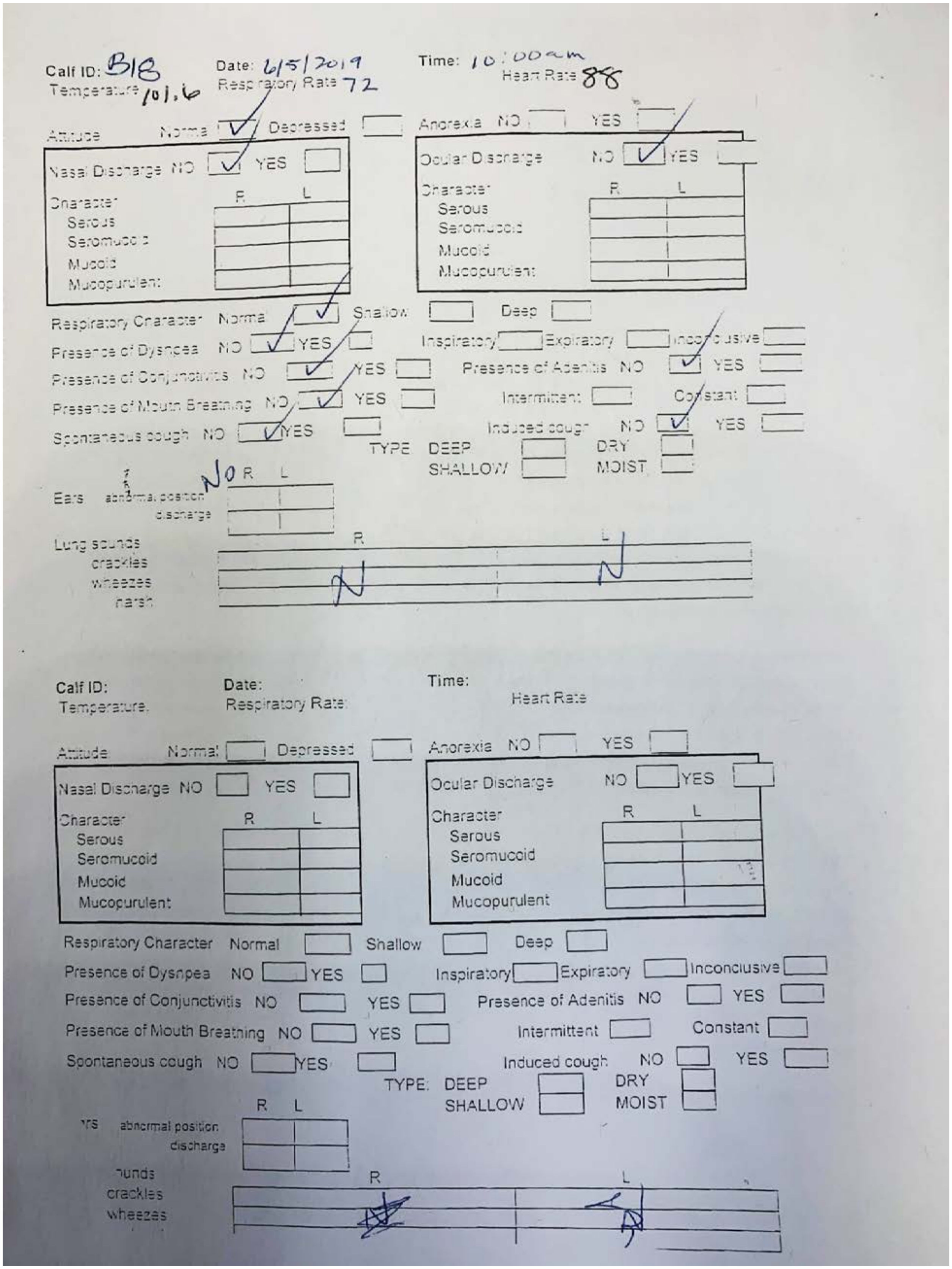

Calf ID: B18 Date: 6/5/2019 Time: 10:00am
Temperature 101.6 Respiratory Rate 72 Heart Rate 88

Attitude Normal ✓ Depressed
Anorexia NO YES

Nasal Discharge NO ✓ YES
Character R L
Serous
Seromucoid
Mucoid
Mucopurulent

Ocular Discharge NO ✓ YES
Character R L
Serous
Seromucoid
Mucoid
Mucopurulent

Respiratory Character Normal ✓ Shallow Deep
Presence of Dyspnea NO ✓ YES Inspiratory Expiratory Inconclusive
Presence of Conjunctivitis NO ✓ YES
Presence of Adenitis NO ✓ YES
Presence of Mouth Breathing NO ✓ YES Intermittent Constant
Spontaneous cough NO ✓ YES
Induced cough NO ✓ YES
TYPE: DEEP SHALLOW DRY MOIST

Ears abnormal position discharge R L — No

Lung sounds crackles wheezes harsh R: N L: N

Calf ID: Date: Time:
Temperature Respiratory Rate Heart Rate

Attitude Normal Depressed
Anorexia NO YES

Nasal Discharge NO YES
Character R L
Serous
Seromucoid
Mucoid
Mucopurulent

Ocular Discharge NO YES
Character R L
Serous
Seromucoid
Mucoid
Mucopurulent

Respiratory Character Normal Shallow Deep
Presence of Dyspnea NO YES Inspiratory Expiratory Inconclusive
Presence of Conjunctivitis NO YES
Presence of Adenitis NO YES
Presence of Mouth Breathing NO YES Intermittent Constant
Spontaneous cough NO YES
Induced cough NO YES
TYPE: DEEP SHALLOW DRY MOIST

[illegible]rs abnormal position discharge R L

[illegible]unds crackles wheezes R L

Supplementary Table S1. Selected plasma samples for BRSV and *M. haemolytica* infections ^{1}H-NMR metabolomic profiling. Each plasma sample, on average, contained 43 ± 8 of the selected metabolites. To avoid the interference of biochemical changes in the plasma due to the use of antibiotics (Ceftiofur) in the interpretation of the NMR metabolomic profile in response to MH only samples designated as Baseline and Infected were used in the univariate and multivariate analyses. For balanced data sets in the multivariate analysis, three samples were used for each calf in each of the baseline and infected states of the MH challenge, Baseline samples were those collected the three days immediately prior to the pathogenic challenge, while infected samples were those collected after pathogenic challenge and the three days prior to starting a regimen of anitibiotics (Cetfiofur 450 mg x 5 days). For BRSV challenged calves, four samples were used from the Baseline and Infected states.

CHALLENGE	Calf's ID	Day	Category	Sample ID	# Identified Metabolites
MH	C_6	D-3	BASELINE	36	48
MH	C_6	D-1	BASELINE	37	58
MH	C_6	D0	BASELINE	38	43
MH	C_7	D-2	BASELINE	39	44
MH	C_7	D-3	BASELINE	40	46
MH	C_7	D0	BASELINE	41	49
MH	C_8	D-2	BASELINE	42	32
MH	C_8	D-1	BASELINE	43	28
MH	C_8	D0	BASELINE	44	36
MH	C_9	D-2	BASELINE	45	43
MH	C_9	D-1	BASELINE	46	37
MH	C_9	D0	BASELINE	47	49
MH	C_10	D-2	BASELINE	48	42
MH	C_10	D-1	BASELINE	49	56
MH	C_10	D0	BASELINE	50	25
MH	C_6	D1	INFECTED	1	49
MH	C_6	D2	INFECTED	2	45
MH	C_6	D3	INFECTED	3	43
MH	C_7	D13	INFECTED	4	51
MH	C_7	D15	INFECTED	5	48
MH	C_7	D17	INFECTED	6	51
MH	C_8	D1	INFECTED	7	43
MH	C_8	D2	INFECTED	8	46
MH	C_8	D19	INFECTED	9	42
MH	C_9	D1	INFECTED	10	33
MH	C_9	D2	INFECTED	11	46
MH	C_9	D3	INFECTED	12	38
MH	C_10	D1	INFECTED	13	40
MH	C_10	D2	INFECTED	14	40
MH	C_10	D3	INFECTED	15	50

CHALLENGE	Calf's ID	Day	Category	Sample ID	# Identified Metabolites
BRSV	C_1	D-3	BASELINE	51	53
BRSV	C_1	D-2	BASELINE	52	35
BRSV	C_1	D-1	BASELINE	53	32
BRSV	C_1	D0	BASELINE	54	48
BRSV	C_2	D-3	BASELINE	55	47
BRSV	C_2	D-2	BASELINE	56	43
BRSV	C_2	D-1	BASELINE	57	51
BRSV	C_2	D0	BASELINE	58	47
BRSV	C_3	D-3	BASELINE	59	56
BRSV	C_3	D-2	BASELINE	60	49
BRSV	C_3	D-1	BASELINE	61	40
BRSV	C_3	D0	BASELINE	62	37
BRSV	C_4	D-3	BASELINE	63	49
BRSV	C_4	D-2	BASELINE	64	48
BRSV	C_4	D-1	BASELINE	65	44
BRSV	C_4	D0	BASELINE	66	47
BRSV	C_5	D-3	BASELINE	67	33
BRSV	C_5	D-2	BASELINE	68	54
BRSV	C_5	D-1	BASELINE	69	41
BRSV	C_5	D0	BASELINE	70	49
BRSV	C_1	D7	INFECTED	16	26
BRSV	C_1	D9	INFECTED	17	28
BRSV	C_1	D11	INFECTED	18	49
BRSV	C_1	D13	INFECTED	19	32
BRSV	C_2	D7	INFECTED	20	47
BRSV	C_2	D3	INFECTED	21	49
BRSV	C_2	D4	INFECTED	22	22
BRSV	C_2	D5	INFECTED	23	42
BRSV	C_3	D7	INFECTED	24	47
BRSV	C_3	D9	INFECTED	25	43
BRSV	C_3	D11	INFECTED	26	39
BRSV	C_3	D13	INFECTED	27	45
BRSV	C_4	D7	INFECTED	28	40
BRSV	C_4	D9	INFECTED	29	42
BRSV	C_4	D11	INFECTED	30	52
BRSV	C_4	D13	INFECTED	31	46
BRSV	C_5	D7	INFECTED	32	44
BRSV	C_5	D9	INFECTED	33	45
BRSV	C_5	D11	INFECTED	34	45
BRSV	C_5	D13	INFECTED	35	48

Supplementary Table S2. Selected ^{1}H-NMR metabolites (n = 72) and their chemical classification. Reviewed from the Bovine Metabolome Database (BMDB)[3], the Livestock Metabolome Database (LMDB)[4], and the Human Metabolome Database (HMDB)[5].

^{1}H-NMR Metabolite	ID	Chemical classification
2-Aminoadipate	1	Alpha amino acid
2-Aminobutyrate	2	L-alpha-amino acid
2-Hydroxybutyrate	3	Alpha hydroxy acids and derivatives
2-Hydroxyisobutyrate	4	Alpha hydroxy acids and derivatives
2-Hydroxyvalerate	5	Hydroxy fatty acid
2-Oxoglutarate	6	Gamma-keto acids and derivatives
3-Hydroxy-3-methylglutarate	7	Dicarboxylic acids and derivatives
3-Hydroxybutyrate	8	Beta hydroxy acids and derivatives
3-Hydroxyisobutyrate	9	Beta hydroxy acids and derivatives
4-Aminobutyrate	10	Gamma amino acids and derivatives
4-Pyridoxate	11	Pyridinecarboxylic acid
5-Aminolevulinate	12	Delta amino acids and derivatives
Acetamide	13	Acetamide
Acetate	14	Carboxylic acid
Acetoacetate	15	Short-chain keto acids and derivatives
Acetone	16	Ketone
Agmatine	17	Guanidine
Alanine	18	Alanine and derivatives
Allantoin	19	Imidazole
Arabinitol	20	Sugar alcohol
Betaine	21	Alpha-amino acids and derivatives
Cadaverine	22	Monoalkylamine
Carnitine	23	Carnitine
Citrulline	24	L-alpha-amino acid
Creatine	25	Alpha-amino acids and derivatives
Creatine phosphate	26	Alpha-amino acids and derivatives
Creatinine	27	Alpha-amino acids and derivatives
Cysteine	28	Cysteine and derivatives
D-Threitol	29	Sugar alcohol
Dimethyl sulfone	30	Sulfone
Dimethylamine	31	Dialkylamine
Ethanol	32	Primary alcohol
Formate	33	Carboxylic acid
Galactitol	34	Sugar alcohol
Glucose	35	Hexose
Glutamate	36	Glutamic acid and derivatives
Glutamine	37	L-alpha-amino acid
Glycine	38	Alpha-amino acid

^{1}H-NMR Metabolite	ID	Chemical classification
Guanidoacetate	39	Alpha-amino acids and derivatives
Histamine	40	2-arylethylamine
Histidine	41	Histidine and derivatives
Isobutyrate	42	Carboxylic acid
Isoleucine	43	Isoleucine and derivatives
Isovalerate	44	Methyl-branched fatty acid
Lactate	45	Alpha hydroxy acids and derivatives
Leucine	46	Leucine and derivatives
Malonate	47	Dicarboxylic acids and derivatives
Methanol	48	Primary alcohol
Methionine	49	Methionine and derivatives
Methylamine	50	Monoalkylamine
N,N-Dimethylglycine	51	Alpha-amino acids and derivatives
N-Acetylglutamate	52	Glutamic acid and derivatives
N-Acetylglycine	53	N-acyl-alpha amino acid
N-Isovaleroylglycine	54	N-acyl-alpha amino acid
N-Methylhydantoin	55	Imidazoline
O-Acetylcholine	56	Acyl choline
Phenylalanine	57	Phenylalanine and derivatives
Propionate	58	Carboxylic acid
Pyruvate	59	Alpha-keto acids and derivatives
Saccharopine	60	Glutamic acid and derivatives
Sarcosine	61	Alpha-amino acid
Succinate	62	Dicarboxylic acids and derivatives
Succinylacetone	63	Medium-chain keto acids and derivatives
Taurine	64	Organosulfonic acid
Threonate	65	Sugar acids and derivatives
Trimethylamine	66	Trialkylamine
Trimethylamine N-oxide	67	Trialkyl amine oxide
Tyrosine	68	Tyrosine and derivatives
Urea	69	Urea
Valine	70	Valine and derivatives
cis-Aconitate	71	Tricarboxylic acids and derivatives
π-Methylhistidine	72	Histidine and derivatives

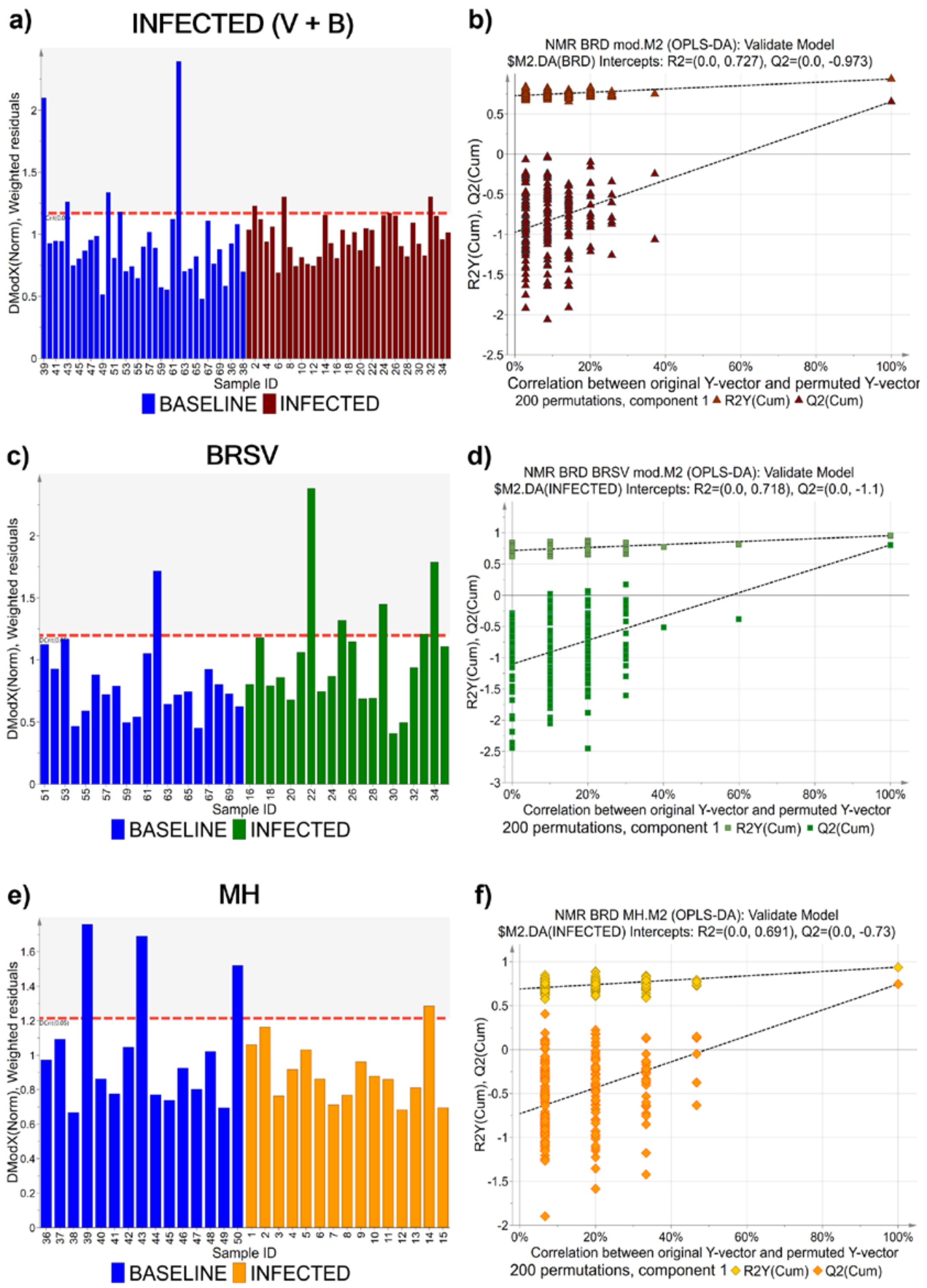

Supplementary Figure S2. OPLS - DA Distance to model plots and Permutation analysis from the viral and bacterial challenge as well as the BRD database. Observations well above the red

line are significantly dissimilar from the others; however, they are not considered outliers if they are not outside the confidence ellipse. (**a**) Baseline vs. Infected (V + B) distance to model plot. Three samples from the Infected category and four from the Baseline can be seen as dissimilar to the others. (**b**) Baseline vs. Infected (V +B) permutation analysis plot. (**c**) Baseline vs. BRSV distance to model plot. Four samples from the Infected category and one from the Baseline are found to be different. (**d**) Baseline vs. BRSV permutation analysis plot. (**e**) Baseline vs. *M. haemolytica* (MH) distance to model plot. One sample from the infected category and four from the Baseline can be seen above the red line. (**f**) Baseline vs. *M. haemolytica* permutation analysis plot.

References

1. Santos-Rivera, M. *et al.* Profiling Mannheimia haemolytica infection in dairy calves using near infrared spectroscopy (NIRS) and multivariate analysis (MVA). *Sci. Rep.* 1–13 (2021). doi:10.1038/s41598-021-81032-x
2. Santos-Rivera, M., Woolums, A. R., Thoresen, M., Meyer, F. & Vance, C. K. Bovine Respiratory Syncytial Virus (BRSV) Infection Detected in Exhaled Breath Condensate of Dairy Calves by Near- Infrared Aquaphotomics. *Molecules* **27**, 549 (2022). https://doi.org/10.3390/molecules27020549
3. Foroutan, A. *et al.* The bovine metabolome. *Metabolites* **10**, 1–26 (2020).
4. Goldansaz, S. A. *et al.* Livestock metabolomics and the livestock metabolome: A systematic review. *PLoS One* **12**, 1–26 (2017).
5. Wishart, D. S. *et al.* HMDB: The human metabolome database. *Nucleic Acids Res.* **35**, 521–526 (2007).
